# Exploring Coping Strategies Among Older Women Who Have Experienced Intimate Partner Violence During COVID-19

**DOI:** 10.1177/10778012231188086

**Published:** 2023-07-28

**Authors:** Christina Safar, Kimberley T. Jackson, Jennifer D. Irwin, Tara Mantler

**Affiliations:** 1 Health and Rehabilitation Sciences Program, Faculty of Health Sciences, 6221 The University of Western Ontario, London, Ontario, Canada; 2 Arthur Labatt Family School of Nursing, Faculty of Health Sciences, 6221 The University of Western Ontario, London, Canada; 3 School of Health Studies, Faculty of Health Sciences, 6221 The University of Western Ontario, London, Canada

**Keywords:** intimate partner violence, coping, COVID-19, older women

## Abstract

This interpretive description study explored coping among older women in Ontario experiencing intimate partner violence (IPV) during COVID-19. Twelve in-depth interviews with older women found age-related normative beliefs played a role in how older women viewed their lives and how they looked beyond their experiences of IPV. Their roles as caretakers and homemakers influenced their response to IPV, and COVID-19 exacerbated feelings of lost time and loneliness. Coping strategies consisted of social support, including telephone formal services and physical activities. Women expressed a lack of appropriate services and financial limitations as barriers. They identified the need for age-appropriate services that acknowledge their unique experiences.

## Introduction

The first case of COVID-19 in Canada was confirmed in January 2020 (Bronca, 2020). Since then, Canada has used public health measures, including a declaration of a state of emergency, physical distancing, and stay-at-home orders to slow the spread of the deadly virus. These public health measures have impacted access to services and coping strategies for all Canadians; however, not all populations have been impacted equally (Lyons & Brewer, 2021). The United Nations has identified that during pandemics and times of trauma, there is a heightened risk for women and girls (United Nations, 2020). One heightened risk for women during the COVID-19 pandemic was the increased prevalence and incidence of intimate partner violence (IPV; Ghoshal, 2020). IPV can be understood as a pattern of physical, sexual, or emotional abuse within the context of coercive control perpetrated by an intimate partner (Tjaden & Thoennes, 2000). Researchers have attributed the increases in IPV to both the public health measures, such as provincial state of emergency and stay-at-home orders, and heightened familial stress (Evans et al., 2020; Gosangi et al., 2021).

IPV is a significant public health concern as it impacts every aspect of women's lives (Ali et al., 2016). In Canada, approximately 44% of women aged 15 and older experience IPV at some point in their lives (Cotter, 2021). There is considerable research on the impact of IPV among younger women (i.e., aged 20–40; Jarnecke & Flanagan 2020); however, there is minimal literature exploring older women's (i.e., 50+ years of age) experiences of IPV and coping. This is of concern as the world is currently facing a major demographic change. Together, the high prevalence of IPV along with the aging population underscores a need to examine experiences of IPV among older adults.

It has been reported that women cope with IPV in many ways. Coping can be understood as the means for managing stress or what individuals perceive as stressful. Understanding coping is important for developing services and programs to assist individuals in dealing with their stressors. However, there is a dearth of literature examining coping and IPV among older adults (i.e., 50+ years of age)^1^. Specifically, there is no research to date focusing on coping strategies of older women experiencing IPV during the COVID-19 pandemic. Prior to the pandemic, there was minimal research and this lack of literature has been attributed to the reality that older women are less likely than younger women to report their experiences of IPV and/or seek formal services (Roberto et al., 2013). Some of the deeply rooted reasons why older women do not seek support include shame and humiliation, fear of having to make a major lifestyle change, guilt about abandoning an abuser in poor health, traditional values of marriage, and the need to keep family matters private (Zink et al., 2003). Research has established that older women's perspectives of patriarchal attitudes gave rise to their partners’ sense of power and privilege which influences whether or not older women will engage with support services (Harris et al., 2012). Together these family norms of abuse and the need to keep experiences of abuse within families decrease the likelihood that older women will engage with support services, meaning many women cope with the abuse on their own; however, little is known about how older women are coping.

Women who experience IPV developed coping strategies that are useful for their contexts (Zink et al., 2003). Coping can be understood as the thoughts and behaviors used to manage the internal and external demands of situations that are appraised as stressful (Folkman & Moskowitz, 2004). While literature regarding coping during COVID-19 for older women experiencing IPV does not yet exist, emotion-focused coping can be understood as strategies used to manage the distress associated with a specific problem; these strategies typically develop over time and are seen as a “philosophy of life” (Folkman & Moskowitz, 2004). A range of emotion-focused coping has been reported by older women experiencing IPV in the United States and Canada including crying, telling someone, becoming more independent, or taking on new activities, the most common strategy was reframing the abuse, for example, their partner may have been physically abusive therefore the emotional abuse they would be facing does not seem as difficult (Divin et al., 2013; Souto et al., 2019; Zink et al., 2004, 2006). Problem-focused coping is typically an action-based strategy designed to deal with the problem (Folkman & Moskowitz, 2004). Older women experiencing IPV identified using problem-focused coping to deal with experiences of abuse which included using routines, care duties, and substances (Lazenbatt et al., 2013; Roberto et al., 2013; Teaster et al., 2006; Zink et al., 2006). By keeping busy and sticking with routines, older women were able to minimize experiences of abuse by trying to appease their abusers. Similarly, another U.S. study of rural older adults experiencing IPV reported older women were able to stay with their abusive partners by focusing on a different problem, which was providing care for their grandchildren (Teaster et al., 2006).

Older women experiencing IPV prior to the pandemic utilized both emotion-focused and problem-focused coping to deal with abuse. However, throughout the COVID-19 pandemic, public health measures have limited what Canadians have been able to do, with research emerging that the coping strategies used prior to the pandemic became severely hampered or overused during the pandemic (Lyons & Brewer, 2021); however, there is currently, no literature examining coping among older women experiencing IPV during the COVID-19 pandemic. As such, the purpose of this study was to explore how older women living in Ontario during COVID-19 coped with their experience of IPV.

## Method

This cross-sectional qualitative study used an interpretive description (ID) framework (Thorne et al., 1997). The interpretive description aims to generate knowledge relevant to the clinical context of applied science (Thorne et al., 2004). Interpretive description was created for nursing studies to break free of the constraints of traditional qualitative methodologies and to build more effectively the knowledge the discipline requires (Beck, 2013; Thorne et al., 2004). This methodology is unique in that it does not follow the rigorous structure of a singular methodology, but rather adopts techniques and skills relevant to that study (Beck, 2013; Thorne et al., 2004). For this study specifically, the main themes of coping and IPV were investigated while considering the uniqueness of each individual situation for each participant. As ID is grounded in action-oriented research, the goal of this analysis was to understand IPV and coping experienced by older women and in turn highlight the needed changes to practice. Within IPV research, ID was useful in determining what types of support this population needed. It is important to note as different generations of populations age their needs will also change.

### Study Procedure

Recruitment for this study was done in the province of Ontario, from March to June of 2021. In 2019 Ontario had a population of 14.6 million people (Ontario Ministry of Finance, 2020), 2.5 million (17.2%) of the population were 65 years of age or older. Recruitment for this study used Kijiji and Facebook advertisements across Ontario, as well as snowball sampling strategies. A total of 1,094 advertisements were posted resulting in the participation of 12 women.

Those eligible to participate in this study participants were (a) women who lived in Ontario, (b) were 50 years of age or older, (c) experienced IPV within the previous year and (d) had access to a safe phone and/or computer. Including women who were 50 years of age or older were selected as this is congruent with the classification of older adults in the IPV literature (Solace Women's Aid, 2016). Participants were eligible if they had experienced any form of physical, emotional, or sexual abuse in the last year (12 months) which was assessed using the four-question abuse assessment screen (AAS) validity tool (Soeken et al., 1998). To determine if participants had access to a safe phone and/or computer they were asked if their devices were safe via a yes or no question. This global question was utilized as previous research in the area has identified that women experiencing violence are experts at knowing how to keep themselves safe and if their devices are safe (Eden et al., 2014; Glass et al., 2015).

#### Data Collection

Data was recorded and collected using semistructured interviews ranging from 24 min to 59 min, averaging 39.8 min. A semistructured approach was selected as it allowed those participating the freedom to express their views in their own words following questions and prompts by the interviewer (Cohen & Crabtree, 2006). All questions were open-ended and focused on coping with IPV during COVID-19. Questions were refined throughout the interview to provide clarifications for participants on terminology and allow for more detailed explanations. The semistructured interview had three parts: (a) context; set the stage for participants’ lives pre- and post-COVID-19 stay-at-home orders; (b) coping and difficulty to cope; looked to understand what strategies had been used by women to manage the stress associated with IPV during the stay-at-home orders in Ontario; and (c) what they needed to improve how they coped. Upon completion of the interview, women were provided with a $15 honorarium in recognition of their time and contributions. After the interview was completed, all recordings were transcribed verbatim by the primary researcher.

#### Data Analysis

The first step of the analysis was to identify each component of the presented data and determine how they are individual and how they worked together; this process continued until there was a sense of clarity rather than an organizational structure (Thorne et al., 1997). Open coding in our study was the process of identifying major themes during the interviews and defining them. Once this was completed, using axial coding, the process of examining the relationship between the emerging themes began to yield insight into the logic and flow of the findings (Thorne et al., 2004). To examine the relationships, the first author went through multiple iterations of coding and made the necessary changes to themes and definitions, then completed a full analysis. The idea was to continually work with the data until it moved from something that was self-evident to that which was not previously known (Thorne et al., 2004). The purpose was to make sense of all the commonalities and variations that were raised within the data (Thorne et al., 2004).

## Results

### Participants

The sample consisted of 12 Canadian women living in Ontario, aged 50–67 (*M *= 53.8) and primarily living in large urban centers (75%, *n* = 9). In total, 58% (*n* = 7) completed postsecondary education, 8% (*n* = 1) had some postsecondary education, and 33% (*n* = 4) completed high school. Average household income (pre-COVID-19) was reported as less than CAN $19,999 by 8% (*n* = 1), CAN $20,000–$49,999 by 33% (*n* = 4), 42% (*n* = 6) reported CAD $50,000 or greater, and 8% (*n* = 1) reported greater than CAN $100,000. Most women identified as living with their partners (50%, *n* = 6), and 58% (*n* = 7) had children. Only 8% (*n* = 1) lived with both their partner and children. Although all participants were required to identify as women to be eligible for this study, gender diversity was observed as 25% (*n* = 3) identified as transwomen. Most women identified as heterosexual, 67% (*n* = 8), 17% (*n* = 2) identified as pansexual, and 17% (*n* = 2) did not specify. See Table 1 for demographic variables.

**Table 1.** Demographics Variables.

Demographic variables	Total
	*n* = 12
Gender	*n* (%)
Women	9 (75)
Transwomen	3 (25)
Education level	
High school	4 (33)
Some college or university	1 (8)
College/university	7 (58)
Indigenous	
Yes	1 (8)
No	11 (92)
Sexuality	
Heterosexual	8 (67)
Pansexual	2 (17)
Not defined	2 (17)
Marital status	
Single	3 (25)
In a relationship not married, common law, or engaged	3 (25)
Married, common law, engaged	3 (25)
Divorced or separated	3 (25)
Household income	
Less than $19, 999	1 (8)
$20,000–49,999	4 (33)
$50,000–99,999	5 (42)
Greater than $100,000	1 (8)
Not defined	1 (8)
Type of community	
Large urban center (100,000 people or more)	9 (75)
Urban center (30,000–99,000)	2 (17)
Rural (30,000 or less)	1 (8)
Children	
Yes	7 (48)
No	5 (42)
Children's living situation	
Live with me full-time	1 (8)
Live with me part-time	1 (8)
Do not live with me	5 (42)
Does not apply	5 (42)
Living situation	
Live alone	4 (33)
Live with partner	6 (50)
Live with partner and children	1 (8)
Other	1 (8)
Essential worker	
Yes	1 (8)
No	11 (92

### Thematic Results

**“**I’m embarrassed, I don’t want to say that this is happening in my home” (Woman (W)3).

Participants described the intersection of age, IPV, COVID-19, and coping. In understanding how coping was influenced by age two subthemes emerged, normative beliefs particularly surrounding traditional gender roles as well as a feeling of limited time. Coping was influenced by COVID-19 as women identified reduced resources, increased experiences of abuse, and pressures to stay with their abusive partners. Women identified coping through social support, physical coping, and online formal coping in the context of their age, IPV, and COVID-19. However, these contextual factors led to barriers in coping.

***Aging.*** Older women described the connection between aging and their experience of IPV through normative beliefs and feeling as though they had limited time. Normative beliefs can be understood as a set of individual beliefs dictating socially what is desirable or appropriate as governed by context (Sprott et al., 2003). Older women identified traditional gender roles including homemaking, caretaking, and being there for others as their responsibility in the relationship and contributing to their normative beliefs about a women's role and place in society. One woman described her responsibility to care for her husband by doing the bulk of the cooking and explained how over time her normative beliefs became a means for her partner to control her, saying:

… a trait we are accommodating. I mean, I do not mind getting up and cooking breakfast before [husband] go to work and having dinner on the table when [he] gets home. Even though I have worked a full day myself. It is how I was raised … I feel a woman … wants to take care of a man but … in other relationships, I have had other expectations. I mean, I do those things, but you are appreciated and you, you do not mind taking the garbage out … it starts that way. And it slowly changes until one day you are trapped in something uh controlling and mean and vicious. (W2)

This role of homemaking and caretaking was repeatedly described as the role of the “women” and it was foundational to her worldview. Women also described the importance of this traditional role of homemaking caretaking as it related to supporting the health of their partners. A woman who had experienced various levels of emotional abuse described the experience of caring for her husband who had multiple comorbidities, and how her partner used this role to further perpetrate abuse, stated:

… My husband, he is not feeling very good. He has diabetes, and he does not really take care of himself. So [he] has kidney problems and heart problems, he has a lot of those and he does not listen. And it is been tough. [He] just have a lot of like, medical kind of checkup I have to help him with and just dealing with my husband at home … but it is not as bad as I think. (W12)

Beyond normative beliefs that governed women's interactions with their partners and everyday lives, most women in this study held beliefs that abuse was a private matter. One Woman described how the signs of abuse were obvious to those around her with friends inquiring about physical injuries. Given her age she felt the need to make up a story to explain the injuries and that no one really pursued the conversation as it is something that is just not talked about. She highlighted how even though people may have assumed she was experiencing IPV she felt the need to not talk about it saying:

… You see like five spots on my arm? … [From] where he grabbed my arm? … Then it will turn purple after two or three days. I [say] I just fell. [Friends would ask] How did you fall like that? … I think they know. But nobody wants to talk to you [about IPV]. (W3)

The normative beliefs that governed their interpersonal relationships contributed to many women staying in the abusive relationship. Another factor contributing to women staying with their abusive partner was many felt they were too old to change their relationship, or it was “too late” for them to leave. Limited time can be understood as feelings of missing out on life and running out of time to resolve their IPV (Band-Winterstein, 2015). One woman described how feeling like it was too late to leave left her feeling embarrassed that she was still in an abusive relationship. This woman explained:

It is embarrassing as well because I should have my shit together. And now I am starting over, you know, so it is kind of humiliating even more so being an older person because you're not where you should be … [younger women] have more time to fix stuff … I have more stress on my plate and next to no time left. (W11)

***COVID-19.*** The older women in this study were experiencing IPV during a global pandemic, which compounded their experiences of violence. Specifically, there was a lack of resources, increased abuse, and pressure to stay with their partners. Women described a lack of resources tailored to their age, during the pandemic. One woman who had utilized a women's shelter during a pandemic expressed hesitancy to do so again as she was frustrated with having to watch and listen to young women and children who were struggling. She explained:

Things are bad for me … I do not want to go to like a, like a woman's violence place, because I do not feel like listening to kids either …. Like I have enough stress without having to listen to kids that are dealing with their losses … trying to figure out how I can fit into whatever to benefit myself, so I can get through it … but if you go into a family shelter you get help faster …. So, you either get tortured with you know mental stress and no privacy … and [if] you cannot handle certain things or noise then it is difficult but at the same time if you bite the bullet you will probably get a home quicker. (W11)

The concern regarding the age of women at shelters was echoed by another woman who said, “there were only three of us over the age of 50, everybody else was 20 or 25 … they had a lot of rules” (W8).

The feeling that services were not designed for the needs of older adults was compounded by COVID-19 stay-at-home order public health restrictions which shut down many services. Women described feeling alone, as many support services shut down during the earlier stages of the pandemic saying, “… no matter what was happening, I felt I had no resources … I had to make the situation as best as I could” (W1). Another woman echoed this saying, “I remember calling (the service line) they said we are closed down … due to the pandemic we have closed down our lines” (W5). When women found the few services that were available, they identified COVID-19-related changes in service meant the resource no longer met their needs. One woman who had accessed a women's shelter during the pandemic, then returned to her partner because of the state of the service said, “I did go to a women's shelter … I was isolated in a bedroom, they brought food to my door … I felt like a prisoner actually … it was horrible” (W2).

Women also underscored how the COVID-19 pandemic, specifically the stay-at-home order from March–June 2020 to April–June 2021 resulted in increased abuse. One woman explained that increased time with her partner created tension resulting in abuse. She said:

(My partner) was not this guy, we were fine (before COVID). We get along so well. And then I think we spent so much time together and it is like, we annoy each other. And I do things like on purpose to annoy him which I admit. I keep the TV loud and he was he would yell at me and he will like drink his beer and throw his beer around and throw the food around says you pick it up. We do (it to) each other. We do things to each other just to irritate each other. I know what pisses him off. He knows what pisses me off. So, everybody presses everybody's buttons, the thing is when he is drunk and he gets very abusive. (W3)

Similarly, another woman attributed the increase in the frequency of abuse to being stuck at home together by stating, “… (before the pandemic) I could get away and he could get away so that he was getting frustrated and abusing me as much he always did, but not so much as when he was stuck at home more” (W10).

Despite the lack of resources available and increased abuse experienced by many women during the stay-at-home orders associated with COVID-19, many older women felt sympathetic to the difficulties that their partners were experiencing during the pandemic. This sense of obligation related to gender norms was why some older women felt pressure to stay with their partners. One woman whose partner lost a loved one during the pandemic described this saying, “… I just wanted this man gone several times now (laughs) but I was there, you know, and (it) was between the pandemic and death in his family and not being able to attend that death. I felt sorry for him” (W9). Another woman pointed out that her partner's medical complications were exacerbated by the lack of resources available during the pandemic which made her feel like she had to stay. She explained:

So back in 2019, I went to a woman shelter in there, I had some counseling, and they, they talked with me too, and they that help. But then I had to come back home, my husband got very sick, and he wanted me to come back home. So I came home. (W12)

***Coping.*** The compounding effects of COVID-19, age, and IPV influenced the ways older women utilized emotion-focused and problem-focused coping strategies. Older women described using emotion-focused coping such as social support, and problem-focused strategies including (a) physical coping and (b) telephone formal coping.

Older women expressed the importance of social support to help with both the pandemic and the abuse. Social support was described by older women as relying on family and friends for comfort and emotional strengthening, it did not necessarily have to be regarding their personal circumstances but having people to talk to help them to feel as though they were not alone. One woman described her social network by saying, “… I mean it is gonna take a lot of time for me to just heal, but other than that just having a strong support network … and being close to my friends or my family knowing that there are other people in my life to fulfill me and bring me happiness … that really means a lot to me” (W4). For some older women, the COVID-19 public health measures such as the stay-at-home orders afforded them the time to engage with their social support systems more. For one woman, the COVID-19 stay-at-home orders increased the time she had at home allowing her to reach out more frequently to her family, something she did not have time for before the pandemic. She explained,

… My family who some are in Pakistan, some are in Ontario, but in a different city, we have been able to FaceTime daily my, my brother, my youngest brother in Pakistan, who has been able to call kind of when it is night for him and morning for us, we all join the group call. So that has been really nice, especially since I am no longer working, I can join. (W12)

Conversely, not all older women had increased access to social coping because of the stay-at-home orders associated with COVID-19, with one woman reporting feeling isolated and having no one to talk to. She said, “I needed an ear to listen … I needed friends … there was no one … maybe having social groups online of women going through the same thing” (W9). Another woman illustrated the importance of social support by explaining how difficult it was for her to cope as she was not seeing her family because of the COVID-19 stay-at-home order. She explained,

I mean, (my mom) supports me over Facebook Messenger and stuff, and we do talk, but it is not the same as having somebody hug you … when you want to talk about emotional pain or emotional struggles, you do not want to do it over the phone. You want to see somebody in person. (W2)

Not being able to connect with friends and family during the pandemic undermined some older women's ability to cope leaving them feeling more isolated. This sense of isolation was underscored by one woman who explained how she was unable to see people she counted on,

It was just a lot more isolating, like, I did not get to see a lot of people. And have not really seen a lot of people, have not seen my daughter tons. She is not in she is in [the same city as me. We know the restrictions there, especially lately …. Just really isolating. And sometimes when things get tense or whatever uh I get a lot of the Oh, the frustration gets taken out a lot on me whether it is just uh attitude or something more. Which is just more than it used to be I guess. (W1)

Physical coping, a form of problem-focused coping, not only helped older women manage everyday stress, but many reported it gave them daily tasks that they looked forward to. Physical coping is understood as action-based strategies that help manage stress and can be associated with doing activities (problem-focused coping) or emotional actions such as crying and screaming as a way to distract the mind (Folkman & Moskowitz, 2004). Popular problem-focused coping activities during the stay-at-home order included cooking and baking as one woman explained, “baking. I mean (laughs), it sounds crazy, but it is my way to escape to escape anything and usually when I am stuck, I mean, even before this relationship, I would bake” (W2).

Another physical coping mechanism mentioned was avoidance. While many coping methods can be seen as a means of avoiding their partners, the purposeful methods of staying out of their partner's way were highlighted by older women. One woman stated she would get out of the way by any means, saying, “… going into my room and reading and he was ranting and raving or something …. Turning the TV on in the other room. Just physically walking away from them” (W10). Another woman took on extra work just to stay out of her partner's way, she said, “I did not want to take on the extra work but then I thought it was a way to run away sort of” (W8). But as many public spaces were closed this avoidance strategy was not always available for all women. One woman noted, “everything is closed, I cannot go hide in the mall. Or I cannot go to Starbucks and sit there for two hours. That makes it difficult to run away” (W6). This need to be able to go somewhere else during COVID-19 was reiterated by many women. Women described the importance of getting out of their houses and being elsewhere. One woman described the refuge she felt when outside saying, “[I] needed to be able to go out [laughs] … even just go into the … whenever I am outdoors, I feel better” (W9).

Many older women talked about the value of formal support offered online as an important coping mechanism. Women underscored the need to have continued access to support services with many of them wanting to be able to speak to someone directly. Being able to speak to someone was important for older women as it meant they were more likely to be directed towards resources that were tailored to their age. One woman spoke of how the assaulted women's helpline was a means of coping for her during the pandemic. She said, “… the women's helpline. They said that they were looking for places for me to live. And they can find me places where like ladies or old people, same situation like myself….” (W5).

***Barriers to coping***. Women used a variety of coping strategies but identified age, lack of financial autonomy, and technology as barriers inhibiting their ability to cope. Women's age impacted the coping mechanisms available to them to deal with the abuse. One woman described the coping mechanism she had previously used to deal with the abuse was no longer a viable option because of her age, saying, “I used to do [physical] activity, but I feel like I am so old now to do activities” (W7). Another woman explained how certain health consequences of abuse, particularly mental health concerns were not spoken about by her age cohort. One woman described that speaking up and getting help as an older woman experiencing IPV was not viewed as acceptable, she stated;

There is no such thing as having anxiety at my age … meanwhile it is a real thing … you have to be quiet about it … younger generations can get help … they know what avenues they have they know more about what their rights are. (W11)

Coping mechanisms were hampered by a lack of financial autonomy. Women described having limited or no access to finances. One woman expressed that having shared finances was a reason for staying with her partner saying, “we bought our home together too I do not know, like we bought a home together. I have not got down payments … I just cannot walk away” (W6). Similarly, one woman highlighted how not having money of her own to rent a place to live was a barrier to leaving. She explained, “I do not have that I just do not have money for first and last (months’ rent)” (W10).

This lack of money and control over finances was further exacerbated by existing social safety nets that did not intersect well with the needs of women because of the COVID-19 pandemic. Some women expressed that they could not even purchase grocery items without their partners, or they could not leave because they did not have the money for rent on their own. To support individuals through financial hardships during the stay-at-home order, the Canadian Emergency Respond Benefit (CERB) was implemented. This benefit was given to eligible participants to offset the effects of lost income due to the stay-at-home orders. In this study, women had other forms of income such as the Ontario Disability Support Program (ODSP), and pensions. Therefore, they were not eligible for CERB funding, despite an increased need for support during COVID-19. One woman underscored this lack of funding support, she said:

I really think that the government needs to do something financially for abuse victims, during the COVID. Anyways, (a woman experiencing IPV) needs to have access, so we can leave, if we want to … (with) no money or no control of the money in your life, it is not like you will walk out the door and go. (W2)

Beyond the financial impact of COVID-19 there was also a significant push for services to adapt to virtual formats such that they could still be offered despite public health guidelines. With these adaptions to virtual formats, there were challenges with the utility of the services and technology barriers for older women. One woman expressed how online services felt impersonal and did not fulfill her need for human connection, she stated, “I do not find them useful. No, I have seen my doctor on the phone. And it was useless … it was frustrating, annoying, it was upsetting (laughs) I did not find it good at all” (W9). Another woman highlighted how older women may not have the ability to operate the online service sphere. She explained:

I am 52. I am pretty old already. And then I have a friend who is 60-years-old who is getting abused. And but she says, I do not know how to use computers and I do not know how to use gadgets. And I do not know how to use like, texting …. She asked me, can you text this my address and my name to this number? I do not know. I do not have I do not have that phone. My phone does not take text. She had like a landline, so she did not know what they were trying to tell her. (W3)

In summary, older women described the normative beliefs surrounding caretaking and homemaking as key factors in their decision to stay with abusive partners. They also described how there was a lack of resources tailored to women their age which inhibited help-seeking. COVID-19 resulted in older women's increased experiences of abuse and added pressure to stay with their abusive partners as support services were operating within strict public health guidelines making them more difficult for women to access and more intolerable than the experiences of abuse themselves. Coping by engaging with social supports, which some women described as easier to do during COVID-19 and others described it as more difficult. Women underscored the importance of physical coping strategies such as hobbies to fill the time and avoiding their partner, as COVID-19 meant they were stuck at home with their partners. Several barriers to coping were also identified including that as they aged some of the coping strategies they had previously used were no longer available, the lack of financial autonomy led them to be stuck in the relationship with this reality being exacerbated by COVID-19, as well as technology barriers in access virtual support services.

## Discussion

The purpose of this study was to explore coping among older women experiencing IPV during the COVID-19 pandemic. Older women felt a duty to be homemakers and caretakers. These roles were viewed by women as both a responsibility and a priority during the pandemic when access to other forms of caretaking and support in the community was limited. Homemaking and caretaking align with socially acceptable roles and normative beliefs about roles that women held that would have been dominant discourses in society when women who participated in this study were being raised (Blackstone, 2003; Sprott et al., 2003). Specifically, Zink et al. (2004) reported similar generational ideologies including traditional religious and family values encouraging roles of homemaking and caretaking. While in the 2003 and 2004 studies mentioned above, women in their 50s would have grown up in the 1950s and 1960s, in our study women in their 50s would have been raised in the 1970s. These differences in time of the age cohorts could account for differences in ideologies and beliefs about the role of women in family settings. These roles of homemaking and caretaking were submissive in the family context, women were to be the support for their spouses and families, which reinforced keeping IPV as a strictly family matter (Band-Winterstein, 2015). Research has established that holding traditional roles, regardless of age, and valuing privacy in family matters are associated with staying in abusive relationships (Band-Winterstein, 2015; Edwards & Dardis, 2020). However, unique to this study were the ways in which older women described how these traditional gender roles were even more problematic in the context of a pandemic, such as being expected to take care of ill partners and maintain their homes as there were no other options for outside help.

Previous research has reported that older women experiencing IPV commonly feel both physical and mental exhaustion and report frustration for time lost (Band-Winterstein, 2015). According to Band-Winterstein (2015), at the end of life, women evaluate events, experiences, and accomplishments which were mostly overshadowed by their experience of IPV. Band-Winterstein (2015) found that during the time in which women experienced IPV, they sensed a time freeze, in which they are focused on the current events in their lives. As end-of-life approached, everything about their lives became more immediate and pressing. Older women then attempted to fulfill themselves and leave something meaningful behind (Band-Winterstein, 2015). Interestingly, the women in our sample, who were 50–67 years of age, expressed similar feelings of lost time as the women in the Brand-Winterstein (2015) study who were 60–84 years of age. The similarities in the feeling of lost time between the two studies could be explained by women in my sample also dealing with the COVID-19 pandemic. It is posited, that being trapped at home gave older women more time to reflect upon their lives, perhaps, exacerbating feelings of aging.

Older women stressed that due to COVID-19, there has been a lack of resources available to them. Additionally, women explained how shelters had restrictions and isolation measures that made existing services unhelpful (Wathen & Mantler, 2022). Specifically, the isolation requirement in shelters and the typically younger demographic using shelters made older women uncomfortable, thus influencing their decisions to return to their abusive partners. While public health measures enacted to contain the COVID-19 pandemic were well-mannered, there were unintended consequences. The public health measures disproportionately disadvantaged certain groups, with women experiencing IPV being one of them (Mantler et al., 2021). Services that would normally be available to support women experiencing IPV were operating at reduced capacity and using alternate digital formats during the pandemic (Lyons & Brewer, 2021). There were also reports of shelters triaging who could use their services, with priority given to those experiencing the most significant forms of abuse and those with children (Mantler et al., 2021). This reduction in available services to women experiencing abuse contributed to women not wanting to engage with existing services out of fear they would not be helpful.

A lack of available services for those experiencing IPV is not unique to the COVID-19 context. In Teaster et al. (2006), lack of resource availability hindered older women's ability to manage the stressors associated with experiencing IPV, further limiting women's ability to reach out for help. Resources that were available had provided online options, but these were not accessible to women who did not have secure devices or for those who felt online means were impersonal. Moreover, prepandemic research by Divin et al. (2013) demonstrated that lack of work experience affected the ability of older women to reach out for help when it comes to IPV, as they did not have the ability to access resources without being surveilled by their partners. The COVID-19 restrictions worked together to decrease the availability of and ability to access the already limited resources for older women experiencing IPV (Mantler et al., 2021).

Another unintended effect of COVID-19 for older women in this sample was increased abuse by their partners. Increased stress leading to amplified abuse was reported by women in this sample, with increased time with partners due to the stay-at-home order being identified as the primary stressor. This is consistent with studies by Gosangi et al. (2021) and Lausi et al. (2021) which found a higher incidence and severity of physical IPV, and an increased prevalence of emotional abuse during the COVID-19 pandemic. The increased abuse was associated with an increase in life stressors such as fear of COVID-19, lost jobs, reduction of finances, impact on social interactions, and other physical and psychological stressors (Lausi et al., 2021). Despite this increased abuse, women stayed with their partners during the pandemic because they felt obligated to care for their partners because of existing physical or mental health conditions.

It has been underscored that older women experiencing IPV tend to use coping strategies that are both social and physical in nature but rely on social and psychological methods such as crying, screaming, and meditating (Zink et al., 2006). Women often seek emotional support from others such as family and friends, imagine their situation as better than it is, set a routine, and establish physical and psychological boundaries (Zink et al., 2006). However, due to the closures of public buildings during the pandemic, including malls, gym facilities, stores, and restaurants, it was noted that older women used emotion-focused coping strategies such as social support, this could be due to the lack of availability of physical-based strategies.

Problem-focused strategies including physical coping strategies are another means of managing stress associated with IPV (Zink et al., 2006). Older women in our study utilized strategies such as baking, cooking, watching TV, doing art activities, and staying out of their partners’ way. Studies prior to COVID-19 regarding physical coping mechanisms of older women found that women relied on routine to help them cope, this includes homemaking, keeping busy with work, volunteering, and exercising (Rizo, 2016; Zink et al., 2006). Closures of public facilities to mitigate the spread of COVD-19 limited women's ability to work, volunteer, and exercise therefore limiting the options they had for coping. Interestingly, in the study by Rizo (2016), it was reported that less common strategies were those that involved artistic expression including but not limited to, drawing, cooking, baking, reading, watching TV, and focusing on pets. Older women in this study reported relying on activities that were artistic in nature. Two reasons could be provided for this difference, the first being this study was conducted during COVID-19 in which activities outside the home were limited. The second reason is the age of the sample. In Rizo (2016), the participants were 18–64 years of age, and in this study, participants were 50–64. It is important to understand when and why women experiencing IPV shift to using artistic methods and how to better support available coping methods during public health restrictions such as stay-at-home orders.

In response to COVID-19 restrictions, many services geared toward women experiencing IPV shifted their operations to online delivery or included online components. According to women in our sample, the benefits of online formal support included having a convenient means of talking to someone as well as being offered assistance with specific concerns. The use of helplines was observed throughout the pandemic for those experiencing IPV. The Vancouver domestic crisis line experienced a 300% increase in calls during the pandemic (Kaukinen, 2020). This suggests that helplines were a viable option for support during the stay-at-home orders.

Women expressed that the financial assistance they were receiving did not sufficiently support them due to increased costs of living. Women also expressed how the resources to support people during COVID-19 did not seem to be geared toward older women. Financial difficulties for older women can be caused by a lack of employment, lower-paying jobs, and a lower pension to support them later on (Hing et al., 2021; Kaukinen, 2020). While the CERB was able to supplement the reduced income for people who were unable to work during the pandemic, many older women were not eligible because their income came from ODSP and pensions. While the amount of money these participants had may not have been directly impacted by the COVID-19 pandemic, the need for financial support stems from the needed autonomy from their partners with whom they have been spending an increased time. Abusers were aware of the financial need due to work closures and/or decreased hours as a result of the pandemic and may have confiscated and/or restricted their access to funds from their partners to exert financial control (Roesch et al., 2020). During COVID-19, older women were at home more which increased their feelings of wanting to leave but their income was insufficient to make this a possibility.

The shift to virtual service options to adhere to COVID-19 restrictions was not without its limitations, particularly for older women in this study. Women experiencing IPV may face structural and practical barriers when accessing digital services while sheltered in place. Older women in this study noted that while they themselves did not struggle with technology, as part of the recruitment for this study was accessing the advertisement online. However, they acknowledged that older women whom they knew have expressed difficulties with technology. They also noted that online services at times felt impersonal. Emezue (2020) outlined other technological issues regarding IPV, including connectivity issues and no technology/low technology situations that would in turn cause accessibility issues. These problems can be further exacerbated in rural communities, among low-income users, and older adults who may be unfamiliar with technology (Emezue, 2020). Similarly, Emezue (2020) also highlighted women's concerns with using digital resources which were found to be impersonal for discussing matters associated with IPV. It is not understood how the transition to online resources impacts women over the age of 65 who are experiencing IPV, and alternative methods for them should be considered during future stay-at-home orders.

The limitations of this research should be considered to contextualize the findings. The recruitment of this sample was via Kijiji and Facebook, which limited the age of the sample to women who were technologically savvy. Given the nature of the study, it is possible that women experiencing more severe forms of IPV during COVID-19 did not participate because of the increased abuse and the coercive control that prevented them from safely accessing devices. Further, this study relied on snowball sampling. As such our sample lacked diversity and was largely Caucasian women, in their early 50s, living in urban locations, and does not reflect the Canadian population. Future studies should use purposive sampling to ensure that participants more accurately reflect the Canadian population. The cross-sectional approach of this study meant that how participants coped was in part a by-product of the stay-at-home order during COVID-19 public health response. An implication for future studies would be to use a longitudinal approach that would have followed participants throughout the changes of rules and regulations and would have captured a more complete picture of how older women coped and responded to COVID-19 and their experience of IPV.

## Conclusion

Older women experiencing IPV during COVID-19 described the role of generational normative beliefs around caretaking and homemaking, in influencing their experiences of abuse and likelihood of staying in the relationship. Older women also described using problem-focused strategies such as artistic coping and avoidance coping strategies as they were compatible with COVID-19 public health restrictions. The emotion-focused coping of social support, particularly engaging with family and friends, was easier for some women during COVID-19 as they had more time, but more difficult for others as their abuser was always around. Older women described the importance of having access to support where they could speak with a person, as this resulted in them being directed to more age-suitable services. Barriers to coping for older women experiencing IPV included that as they age some of their previously used coping mechanisms were no longer available (i.e., physical activity). Women also identified a lack of financial autonomy and technological barriers as inhibitors to leaving and accessing resources, respectively.

This study was able to identify women's feelings regarding online coping strategies and formal resources while reiterating the importance of social support. This study also highlighted how COVID-19 restrictions challenged the existing coping strategies typically used by older women and were compounding to their IPV experience as they spent more time with their partners. A gap in existing services, specifically the use of technology and age-appropriate services, was highlighted which resulted in important recommendations including considering the needs of women experiencing IPV when deciding on public health measures and deeming women's shelters and other services as essential. Public awareness about how IPV intersects with family and the roles of older women and public education on IPV and how to recognize and discuss it with family and friends. Other recommendations include domestic violence should consider tailoring resources to meet the needs of older women, and the government should consider providing subsidies and financial resources so women can leave IPV relationships without financial barriers.

Older women found existing resources, such as women's helplines, to be useful to them during the stay-at-home order. Yet, these services did not consider that older women have ideologies that are deeply rooted in traditional gender roles and feeling the need to care for a partner makes it difficult for them to leave the abusive relationship. It is important that service providers understand that older women experiencing IPV have found ways to cope that are directly related to traditional women's roles. Service providers should recognize that homemaking and caretaking is a priority for some older women; therefore, finding ways to support older women in either leaving these roles within their relationship or fulfilling them so they can leave.

Coping strategies for IPV among older women were heavily reliant on social and emotional support. IPV in older age can be difficult as women become physically frail and increasingly exhausted. Being able to identify signs of psychological and emotional IPV among older women and ensuring they feel heard can impact how these women feel about themselves and generally improve their outlook on life. Social and emotional support needs to not only be provided online but also in person. In-person social interactions allow women to be able to escape their physical environment. Financial support was also important for older women experiencing IPV as older women may stay in abusive relationships due to a lack of available finances. Older women who live off of subsidies or pensions were not making a sufficient amount of money and may lack savings. These financial disparities coupled with IPV limited older women's ability to cope and were a major reason they stayed.

Research has been robust in trying to uncover the IPV experiences, consequences, and ways of coping among young women but has lacked focus on older women. Assuming homogeneity in IPV experiences results in critical gaps in understanding, gaps that this study sought to fill. IPV can be experienced by women at any age, however, there is a cumulative effect that can exist as women age (Roberto et al., 2013). Further research is needed on older women's experiences, particularly their ability to cope when specific coping strategies become unavailable, how differing generational beliefs influence the coping strategies, and the integration of evidence to formal supports designed to the meet the needs of older women experiencing IPV, both during pandemics and beyond.

## Notes

1. The term “older adults” are defined as those who are 50 years of age and older.
